# Regulation of Trafficking and Signaling of the High Affinity IgE Receptor by FcεRIβ and the Potential Impact of FcεRIβ Splicing in Allergic Inflammation

**DOI:** 10.3390/ijms23020788

**Published:** 2022-01-12

**Authors:** Greer K. Arthur, Glenn Cruse

**Affiliations:** 1 Department of Population Health and Pathobiology, College of Veterinary Medicine, NC State University, Raleigh, NC 27607, USA; gkarthur@ncsu.edu; 2 Department of Molecular Biomedical Sciences, College of Veterinary Medicine, NC State University, Raleigh, NC 27607, USA

**Keywords:** mast cell, IgE receptor, FcεRIβ, antisense therapy, allergy, asthma, exon skipping

## Abstract

Mast cells are tissue-resident immune cells that function in both innate and adaptive immunity through the release of both preformed granule-stored mediators, and newly generated proinflammatory mediators that contribute to the generation of both the early and late phases of the allergic inflammatory response. Although mast cells can be activated by a vast array of mediators to contribute to homeostasis and pathophysiology in diverse settings and contexts, in this review, we will focus on the canonical setting of IgE-mediated activation and allergic inflammation. IgE-dependent activation of mast cells occurs through the high affinity IgE receptor, FcεRI, which is a multimeric receptor complex that, once crosslinked by antigen, triggers a cascade of signaling to generate a robust response in mast cells. Here, we discuss FcεRI structure and function, and describe established and emerging roles of the β subunit of FcεRI (FcεRIβ) in regulating mast cell function and FcεRI trafficking and signaling. We discuss current approaches to target IgE and FcεRI signaling and emerging approaches that could target FcεRIβ specifically. We examine how alternative splicing of FcεRIβ alters protein function and how manipulation of splicing could be employed as a therapeutic approach. Targeting FcεRI directly and/or IgE binding to FcεRI are promising approaches to therapeutics for allergic inflammation. The characteristic role of FcεRIβ in both trafficking and signaling of the FcεRI receptor complex, the specificity to IgE-mediated activation pathways, and the preferential expression in mast cells and basophils, makes FcεRIβ an excellent, but challenging, candidate for therapeutic strategies in allergy and asthma, if targeting can be realized.

## 1. Introduction

Chronic allergic diseases affect approximately 300 million people worldwide [1], and are often the result of inappropriate, detrimental immune responses to typically harmless environmental antigens. Chronic allergic diseases have complex pathophysiology involving interactions between many immune cells. Of these cells, an important population are mast cells, which play a key role in triggering the immediate allergic response and likely drive allergic inflammation through direct interactions with immunoglobulin E (IgE). Mast cells originate from the bone marrow and are derived from CD34^+^ and CD117^+^ pluripotent hematopoietic stem cells. Initially, they circulate in the blood as committed progenitors, and are then recruited into tissues where they mature and terminally differentiate into mast cells (for review see [2]). Mast cells are present throughout mucosal and connective tissues, where they monitor the local tissue environment and are believed to act as sentinel cells (reviewed in [3]). Despite their relationship with allergic diseases, mast cells are conserved across vertebrate species, and mast cell deficiency in humans is not known to exist. Consequently, mast cells and IgE are thought to have an evolutionary advantage, potentially by participating in type 2 immune responses to parasites and protecting the host against noxious substances such as venoms (discussed in [4]).

Tissue-resident mast cells express the high affinity IgE receptor, FcεRI, on their surface. FcεRI is a multichain immunorecognition receptor (MIRR) that binds to monovalent IgE with very high affinity *K_a_* = 10^10^–10^11^ M^−1^ that is orders of magnitude above that of IgG binding to any of the FcRs [5]. Despite the association rate of *k_on_* ≈ 10^5^ M^−1^ s^−1^ for IgE binding with FcεRI being comparable with IgG associating with its high affinity receptor, FcγRI, the dissociation rate for IgE (*k_off_* ≈ 10^−5^ s^−1^) is at least an order of magnitude slower than that of IgG [5]. Therefore, the result of the slow off-rate for IgE is an increased half-life and prolonged presence of IgE on the cell surface, compared to other immunoglobulins. IgE has a well-established central role in allergy [6,7,8] and is produced by B cells following antigen presentation to naïve T cells. Unbound IgE circulates in serum with a half-life in blood of between 2–4 days [9]. However, upon binding to FcεRI, IgE forms a stable IgE-FcεRI complex resulting in increased surface expression of both FcεRI and IgE due to reduced endocytosis and degradation of FcεRI rather than upregulation of expression by increased synthesis [10,11,12]. Therefore, binding of IgE to FcεRI results in surface IgE that persist on mast cells for prolonged periods, likely contributing to the calculated half-life of IgE in tissues being much greater than blood (16–20 days) [9]. This process of loading FcεRI with IgE is known as sensitization, and primes mast cells and basophils to react to multivalent antigens via the IgE-FcεRI complex. Upon activation, mast cells and basophils rapidly release inflammatory mediators such as histamine, proteases and lipid eicosanoids, which constitute a major component of the acute “early-phase” allergic response [13,14]. Signaling via IgE-FcεRI complexes also contributes to the development of a “late-phase” allergic response by initiating the synthesis and secretion of proinflammatory cytokines and chemokines, which recruit and activate other key inflammatory effector cells, such as eosinophils and T cells (reviewed in [7,13]).

## 2. FcεRI Structure and Function

**Figure 1:**
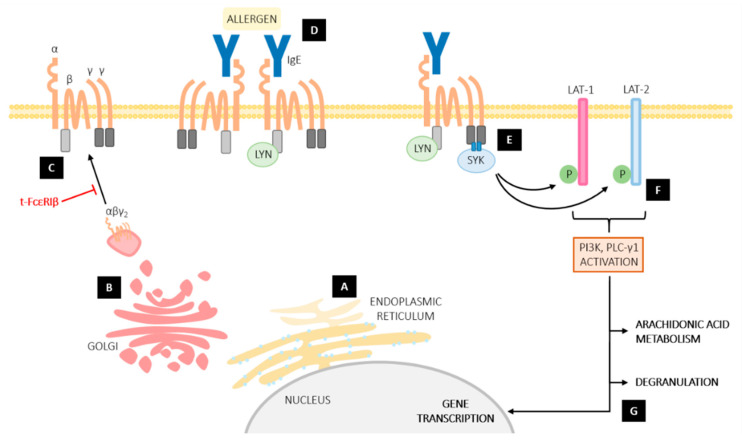
The role of FcεRIβ in mast cell signaling pathways. (**A**) Synthesis of FcεRI α, β and γ-subunits takes place within the endoplasmic reticulum. FcεRIβ facilitates appropriate glycosylation and folding of FcεRIα, and the γ-subunits permit export of the αβγ2 tetrameric complex to the Golgi. (**B**) Full-length FcεRIβ traffics the receptor complex to the cell surface, whereas t-FcεRIβ, which is incapable of trafficking to the surface, prevents surface expression of FcεRI. (**C**) Once at the surface, full-length FcεRIβ stabilizes the receptor complex. (**D**) Binding of IgE to the receptor increases receptor half-life at the surface. Allergen binding cross-links multiple FcεRI and induces receptor aggregation, which leads to phosphorylation of FcεRIβ by LYN. (**E**) By binding LYN, FcεRIβ amplifies phosphorylation of the FcεRIγ ITAMs, which leads to the recruitment and phosphorylation of SYK. (**F**) Phosphorylated SYK propagates intracellular signals by phosphorylating LAT and LAT2, which subsequently induce PI3K and PLC-γ1 signaling cascades. (**G**) Ultimately, these signaling pathways culminate in proinflammatory gene expression and the release of cytokines and chemokines, arachidonic acid metabolism and eicosanoid production, and mediator release via degranulation. By preventing trafficking of FcεRI to the surface, t-FcεRIβ inhibits the downstream cellular events of SYK phosphorylation, including mast cell mediator release. LYN, SRC family protein tyrosine kinase; ITAM, immunoreceptor tyrosine-based activation motifs; SYK, spleen tyrosine kinase; LAT-1, linker for activation of T cells; LAT-2, LAT-1 related adaptor; PI3K, phosphatidylinositol 3-kinase; PLC-γ1, phospholipase C-γ1.

The canonical composition of FcεRI is that of a tetrameric receptor complex with a total of seven transmembrane regions within the complex (Figure 1). The tetrameric form of the receptor complex consists of one α-subunit (FcεRIα) that contains a single transmembrane α-helix; one β-subunit (FcεRIβ) that contains four transmembrane α-helices and cytoplasmic amino and carboxyl termini; and a dimer of two disulphide linked γ-subunits (FcεRIγ) that each contain a single transmembrane α-helix [15]. FcεRIα comprises of a large extracellular portion that contains two immunoglobulin superfamily domains, termed α1 and α2, followed by a transmembrane helix and a short cytoplasmic domain that does not contain signaling capacity [15]. Due to the nature of the FcεRI complex, the three-dimensional structure of the entire complex is unknown. However, the extracellular domains of FcεRIα have been studied by using a recombinant soluble FcεRIα (sFcεRIα) protein to generate a crystal structure [16]. This structure demonstrates that the extracellular FcεRIα domains are responsible for binding IgE and dynamic conformational changes in IgE and FcεRIα play important roles in the interaction (For review see [5]).

Mast cells are not the only cell type that express FcεRI. In humans, the receptor is also expressed by basophils, dendritic cells, eosinophils, monocytes, neutrophils, platelets and smooth muscle cells [17,18,19,20,21,22,23,24]. However, a key difference between these cell types is whether FcεRI exists as a trimeric αγ_2_ or tetrameric αβγ_2_ complex, and whether the receptor complex is constitutively expressed or inducible. Mast cells and basophils are known to constitutively express FcεRI and express the tetrameric αβγ_2_ form of the receptor. The presence of FcεRIβ and the function of the FcεRI complex is less clear for immune cells outside of the mast cell and basophil compartments. Dendritic cells, neutrophils, eosinophils, monocytes and other cells can be induced to express FcεRI that are either in the tetrameric αβγ_2_, or trimeric αγ_2_ configurations [17,18,19,20,21,22,23,24] (reviewed in [25]). However, while it is clear that FcεRI complexes containing or lacking FcεRIβ can be expressed at the cell surface, particularly in humans, it is not yet clear which cell types express FcεRIβ protein, if it has a conserved function in cells outside of mast cells and basophils, or whether expression can change depending upon inflammatory conditions.

Although both αγ_2_ and αβγ_2_ complexes are capable of binding IgE with high affinity, the differential expression of tetrameric αβγ_2_ and trimeric αγ_2_ FcεRI on distinct immune cell populations suggests that the two configurations of the receptor have divergent roles in immunity. In mast cells, FcεRI-mediated cell activation and the distinct roles of each subunit of the tetramer have been well characterized [25] (Figure 1). The extracellular portion of FcεRIα belongs to the immunoglobulin superfamily and binds the Fc portion of IgE [26]. The two γ-subunits comprise the main signaling substructure of the receptor, and together form a signal-transducing disulphide-linked homodimer. In their cytoplasmic domains, the γ-subunits contain immunoreceptor tyrosine-based activation motifs (ITAMs); following antigen cross linking of FcεRIα-bound IgE, signaling is initiated by phosphorylation of each ITAM’s tyrosine residues that recruit and activate the dual SH2 domain-containing, non-receptor tyrosine kinase SYK [27,28]. FcεRIβ facilitates signaling by binding SRC family kinases such as LYN via its own, non-canonical ITAM, located near its C-terminus. This acts as an activation loop [29,30,31], which subsequently leads to recruitment and phosphorylation of SYK from the cell cytosol [32,33] The mechanism of SYK recruitment and phosphorylation appears to be modulated by the kinetics of FcεRI aggregation, since the aggregation of larger numbers of IgE-FcεRI complexes provides a greater pool of phosphorylated ITAMs for SYK to bind to [34,35]. The formation of FcεRI aggregates (which can differ in the orientation and distance of the receptors within aggregates) appears to modulate signaling efficiency, as well as negative regulation of FcεRI activation by the preferential recruitment of inhibitory phosphatases over SYK [36,37]. Correspondingly, the phosphorylation kinetics of SYK, regulated by the duration of ITAM binding, dictates SYK-mediated cellular outcomes [34].

Like other MIRRs, such as the B cell receptor and T cell receptor, FcεRI lacks intrinsic kinase ability, so recruitment of kinases such as SYK by its γ-subunits is critical for FcεRI signal transduction [38,39]. FcεRI-mediated SYK activation propagates intracellular signals by activating phospholipase C (PLC)-γ1, which induces the release of free calcium ions (Ca^2+^) from intracellular stores via the generation of the intracellular secondary messenger inositol triphosphate (IP_3_). Depletion of these stores leads to activation of the calcium-release activated ion channel Orai1 within the plasma membrane, which opens to allow Ca^2+^ influx [40,41,42]. Through spatiotemporally distinct Ca^2+^ fluxes, Orai1 activity triggers a variety of calcium-dependent events, including gene expression (reviewed in [43]), exocytic release of proinflammatory mediators and eicosanoid production, as well as sustainment of the receptor-triggered SYK-mediated signal (for reviews, see [44,45,46,47,48]). FcεRI activation also triggers the phosphatidylinositol 3-kinase (PI3K) pathway in mast cells, which amplifies PLC-γ1-mediated signaling and may also contribute to cell survival and growth (reviewed in [49]).

In comparison to the tetrameric isoform, the properties and roles of trimeric FcεRI are more obscure—a consequence, in part, of differences between humans and mice. Human FcεRIα possesses an extracellular domain that facilitates trafficking with the γ-subunits to the cell surface in the absence of the β-subunit [50], whereas mouse α and γ-subunits will only traffic to the cell surface if the β-subunit is present [51]. In contrast to the pro-inflammatory role of tetrameric FcεRI on mast cells and basophils, studies suggest that trimeric FcεRI expressed on antigen-presenting cells may have an immunomodulatory role (reviewed in [52]), by both restraining allergic tissue inflammation [53] and reducing serum IgE [54]. Whether the proposed anti-inflammatory role of trimeric FcεRI is due to its lack of the signal-amplifying β-subunit is unclear, but the association of the two FcεRI isoforms with distinct patterns of SYK phosphorylation and signaling in different cell types warrants further study of FcεRIβ in the modulation of anti- and pro-inflammatory cell signaling (discussed in [53]). Other factors that could regulate these processes and may hinder research into different FcεRI complexes that exist in human and mouse cells may reside in the tissue microenvironment, where human studies become more difficult to replicate in vitro. In such a setting, perhaps utilizing innovative extracellular matrix-based models, which create a more physiologically relevant environment for cells in vitro could help overcome shortcomings of rodent models [55]. However, even given these shortcomings, it is clear that, in mast cells and basophils, the unique role of FcεRIβ in cell activation and pro-inflammatory mediator release makes it a relatively cell-specific target, and one with the potential to dampen mast cell responses in allergic inflammation.

## 3. Existing Treatments Targeting FcεRI and IgE

As key effector cells in allergy and inflammation, mast cells and basophils are ideal therapeutic targets. Additionally, due to the high affinity binding of IgE to FcεRI, thereby allowing the cells to be primed for activation for months [56], inhibiting or blocking IgE binding to FcεRI receptors is a logical strategy for dampening mast cell and basophil activation. An effective treatment that directly targets this mechanism is omalizumab, a monoclonal anti-IgE antibody that sequesters circulating serum IgE and accordingly attenuates IgE-mediated responses to allergens. Omalizumab, and related antibody therapies, block binding of IgE to FcεRIα, which in turn depletes free IgE from the blood [57]. The result is a gradual reduction in antigen-specific IgE bound to FcεRIα and reduced capacity for mast cells and basophils to respond to allergens. The effectiveness of this mechanism has led to omalizumab becoming an important therapeutic option for antihistamine-resistant diseases, such as chronic urticaria [58] and severe asthma with elevated serum IgE [59,60]. Importantly, in moderate to severe asthma, omalizumab has clinical benefits and facilitates the withdrawal of concomitant steroid and bronchodilator treatments [59,60,61,62]. Some efficacy is also seen in other IgE-driven diseases, such as allergic rhinitis [63], food allergy [64,65], atopic dermatitis [66,67] and urticaria [68,69,70] as well as in patients with multiple allergic comorbidities [71].

Despite these significant benefits, omalizumab, for a variety of known and unknown reasons, can have variable efficacies in different allergic disease settings and their patient subsets [72], (reviewed in [73]). Additionally, benefits arising from treatment with omalizumab can take several weeks to be observed in a clinical setting [74,75,76]. A key factor that makes omalizumab and related therapies effective is the simultaneous decrease of both surface IgE-FcεRIα complexes and FcεRIα surface expression [57]; however, this decrease in FcεRIα expression is markedly slower in mast cells than in basophils. The reason for this difference is unclear, but may be a result of the shorter lifespan of basophils, compared to mast cells, or may be due to other factors, such as differences in IgE half-lives on the surface of the cells [57]. Additionally, the long half-life of IgE-FcεRI complexes also limits the rate of action of therapeutics that aim to block IgE binding [77].

Other limitations to the approach of preventing IgE from binding to FcεRI include the intrinsic sensitivity of mast cells and basophils to IgE-mediated stimulation, which makes it challenging to reduce IgE levels at disease sites to therapeutically beneficial concentrations [73]. Not only is cellular sensitivity to IgE-mediated activation highly variable, but FcεRI is expressed on mast cells in surplus. Despite needing only a few hundred receptors to initiate degranulation [78], human lung mast cells, for instance, may express as many as 130,000 FcεRI per cell [79]. Consequently, if less than 5% of IgE is sufficient to activate the small proportion of FcεRI receptors on mast cells required for a degranulation response, reducing serum IgE by more than 95% could be ineffective in some instances (discussed in [73]). The amount of antigen specific IgE in the pool of total IgE, as well as the valency of the antigen, could also contribute to variable efficacy in different settings. While these factors are not the only possible explanations for the variable efficacy of omalizumab, they demonstrate the inherent difficulty of suppressing IgE-mediated allergic mast cell and basophil activation. Progress may be made by improvements in binding affinity, such as with ligelizumab, which binds to the same constant (Cε3) domain of free IgE with higher affinity than omalizumab and may prove more efficacious in IgE-mediated diseases and chronic spontaneous urticaria [80,81,82].

Anti-FcεRIα antibodies could also have greater clinical efficacy than omalizumab in patients with high serum IgE levels, since they compete with serum IgE for receptor sites and thus act more like competitive inhibitors [83]. In in vivo studies, for instance, monoclonal antibodies targeting FcεRIα suppress anaphylaxis more rapidly than either omalizumab or ligelizumab [84]. Although IgE binding stabilizes expression of FcεRI at the cell surface, thereby increasing the number of receptors capable of binding IgE (reviewed in [85]), allergen-induced receptor aggregation can induce endocytic internalization of FcεRI. Ubiquitination facilitates this process, and eventually leads to lysosomal degradation of the receptor [86]. By triggering aggregation and receptor internalization, anti-FcεRIα antibodies reduce surface FcεRI and render mast cells unresponsive [83]. However, as an anti-allergy therapy, there are restrictions to this approach. Anti-FcεRIα antibodies would be limited to allergen desensitization rather than suppression of established IgE-mediated mast cell inflammation, because existing anti-FcεRIα antibodies are unable to bind to FcεRI if an IgE molecule is already bound [83]. Furthermore, the effectiveness of these antibodies, and other comparable methods of desensitization [72,87], are still limited by the potent ability of small numbers of the receptor to activate mast cells—a challenge shared by other IgE inhibitors, including peptides [88,89,90], oligonucleotide ligands [91] and designed ankyrin repeat proteins (DARPins) [92,93]. Nonetheless, studies examining the dissociative effect of DARPins demonstrate that targeting IgE-FcεRI complexes could help combat allergic responses by not only reducing serum IgE, but also accelerating dissociation of IgE from FcεRI and interrupting the allergic signaling cascade [94,95].

Co-engagement of FcεRI and its inhibitory receptor, the low affinity Fc receptor FcγRIIb, is an alternative tactic to inhibiting FcεRI-mediated inflammation. Unlike the ITAMs of FcεRIβ and FcεRIγ, the cytoplasmic tail of FcγRIIb contains an immunoreceptor tyrosine-based inhibitory motif (ITIM). When co-aggregated with FcεRI, phosphorylation of this ITIM leads to recruitment of SH2 domain-containing inositol 5-phosphatase (SHIP). SHIP recruitment inhibits FcεRI activation and calcium influx [96,97] by preventing Bruton’s tyrosine kinase (BTK) recruitment and subsequent PLC-γ1 activation by SYK [98], (reviewed in [99]). By utilizing this inhibitory mechanism, bi-specific molecules capable of simultaneously binding both FcεRI and FcγRIIb reduce IgE-mediated mast cell degranulation and could become effective therapies for allergic diseases [100,101,102,103].

Inhibiting intracellular signals, such as tyrosine kinase inhibitors targeting SYK or modulating Raf kinase inhibitor protein (RKIP) activity, represent other potential options for allergic therapeutics, due to their roles in IgE-mediated mast cell activation [104,105,106]. The receptor tyrosine kinase KIT, which drives cell cycle progression, DNA synthesis and cell division in mast cells and basophils, may also be a useful target. Importantly, although inhibiting these kinases suppresses mast cell survival and mast cell-mediated inflammation [107,108,109,110], their cell-specificity must be examined, as well as the effect of mast cell and basophil ablation on innate and adaptive immunity (discussed in [14]).

## 4. Alternative Splicing of FcεRIβ and the Functions of Splice Variants

The above discussion demonstrates that efforts to therapeutically suppress FcεRI-mediated mast cell activation are ongoing and a need remains for alternative approaches. To this end, FcεRIβ presents a potentially appealing target. In addition to its role in amplifying IgE-mediated mast cell activation, polymorphisms in the gene encoding FcεRIβ, *MS4A2*, have been linked to allergy and asthma susceptibility [111,112,113,114,115], suggesting a potential role for FcεRIβ in development of allergy. The idea of FcεRIβ as a therapeutic target is not new, but also not straightforward. The clinical benefits of targeting FcεRIβ have been ambiguous, since the association of *MS4A2* polymorphisms with allergy and asthma is not consistent [116,117,118] and transfection of *MS4A2* cDNA containing mutations associated with asthma has previously failed to alter FcεRIβ function [119,120]. The implications of other polymorphisms in the predicted transcription promoter region and in exon 7 of *MS4A2*, which are linked to asthma susceptibility, remain elusive [121,122,123].

**Figure 2:**
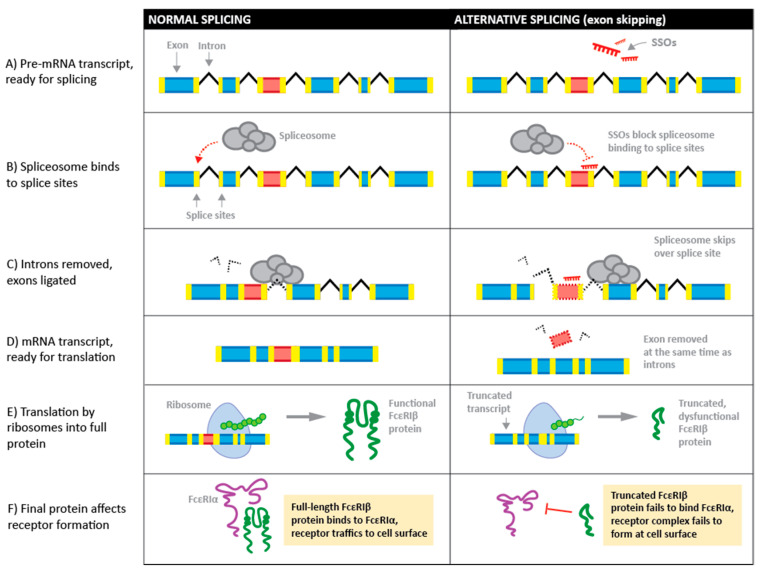
Process of alternative splicing to produce truncated FcεRIβ using splice switching oligonucleotides (SSOs).

Although initial attempts to associate polymorphisms in *MS4A2* with functional outcomes in disease were unsuccessful, the subsequent elucidation of alternative splicing of FcεRIβ mRNA and the functional differences of alternate isoforms have established new avenues of study and reignited interest in the therapeutic potential of FcεRIβ. Alternative splicing enables a single gene to generate a variety of different mRNA transcripts and protein isoforms and is thereby an important regulatory component of eukaryotic gene expression [124]. Splicing occurs when the spliceosome, a complex comprising five small nuclear ribonucleoprotein subunits and various protein cofactors [125], recognizes a splice site within the pre-mRNA transcript. During splicing, the spliceosome catalyzes the removal of introns before ligating the remaining exons to produce a continuous mRNA message (Figure 2). Despite the fundamental importance of alternative splicing in eukaryotic cells, alternative splicing of genes and the effects of polymorphisms on regulating splicing mechanisms are often overlooked and studies of expression in disease states do not always take alternative splicing into consideration. Overall, mRNA transcript number may not change, but skewed splicing could be present and may markedly alter the gene function. FcεRIβ is an excellent example of how apparently minor alterations in transcripts by alternative splicing can markedly affect protein function and regulate processes that would be missed if not specifically examined.

In addition to its ITAM-mediated signaling capacity, FcεRIβ performs the crucial role of trafficking FcεRIα to the plasma membrane, which takes place after the two subunits associate during an early stage of biosynthesis in the endoplasmic reticulum [119,126]. Association of the α and β chains facilitates glycosylation and folding of the α chain, and the arrival of the γ chains permits export of the αβγ_2_ receptor complex from the endoplasmic reticulum (reviewed in [25]). In particular, the first transmembrane helix of FcεRIβ is critical for trafficking and stabilizing the receptor complex, ultimately increasing FcεRI surface expression [51,119,127]. Binding of IgE to FcεRIα adds further stability to the receptor and prevents its internalization [25]. Since the ability of FcεRIβ to fulfill these functions depends upon its polypeptide sequence and structure, alternative splicing of the *MS4A2* gene represents a critical regulatory mechanism of FcεRIβ expression and function.

In human basophils and cord blood-derived mast cells, a truncated splice variant of *MS4A2* (*MS4A2* variant 2-FcεRIβ_T_) caused by inclusion of intron 5 results in a premature in-frame stop codon and loss of the downstream third and fourth transmembrane regions, as well as the C terminal ITAM [128]. Since the first transmembrane helix of FcεRIβ is sufficient to bind FcεRIα and traffic the FcεRI complex [127], FcεRIβ_T_ can associate with FcεRIα. However, FcεRIβ_T_ competes with full-length FcεRIβ for FcεRIα binding, and redirects FcεRIα to endosomes and proteasomal degradation rather than to the plasma membrane [128]. Consequently, the relative abundance of each splice variant determines the proportion of FcεRIβ capable of trafficking FcεRIα to the plasma membrane or through degradation pathways, and thus alternative splicing modulates FcεRI surface expression [128].

Human mast cells also express *MS4A2* variant 3, which excludes exon 3 and produces a truncated isoform (t-FcεRIβ) that lacks the first two transmembrane regions [129]. The first transmembrane helix is necessary for binding to FcεRIα and trafficking of the receptor complex [127], and thus loss of exon 3 restricts t-FcεRIβ to the cytoplasm, nuclear membrane and juxtanuclear organelles [129]. Adenoviral transduction of high levels of t-FcεRIβ triggers cell death in human lung mast cells, inhibits proliferation and induces apoptosis in the rapidly dividing mast cell line HMC-1 [129]. However, *MS4A2* variant 3 also plays a role in IgE-mediated mast cell activation. Following mast cell activation, t-FcεRIβ interacts with calmodulin via a putative calmodulin-binding domain and, most likely through its C-terminal ITAM, also binds Fyn kinase, GRB2-associated-binding protein (Gab)-2, and the phosphoinositide 3 kinase (PI3K) p85 subunit [130]. Together, these interactions enable translocation of t-FcεRIβ to the Golgi, where it facilitates the formation of microtubules that are required for FcεRI-induced granule translocation to the plasma membrane, prior to exocytosis [130,131].

## 5. Functional Outcomes of Modulating FcεRIβ Expression in Mast Cells

By determining the various roles of *MS4A2* splice variants in mast cells, it becomes apparent that alternative splicing of FcεRIβ pre-mRNA selectively removes domains in the protein that are critical for specific functions. Therefore, in addition to regulation of FcεRI expression by transcription of the FcεRI subunits, alternative splicing of FcεRIβ pre-mRNA may regulate FcεRI trafficking to the cell surface, since alternative splicing dictates the intracellular trafficking and function of FcεRIβ. Thus, altered splicing of *MS4A2* could have implications in susceptibility to allergic diseases. Moreover, manipulation of *MS4A2* splicing to favor a particular phenotype, such as aberrant FcεRIα trafficking, could have therapeutic potential.

To manipulate splicing of *MS4A2*, splice switching oligonucleotides (SSOs) targeting exon 3 of *MS4A2* (herein referred to as FcεRIβ SSOs) have been employed by our group [132]. SSOs are beginning to show promise as therapeutics in personalized medicine. They consist of short synthetic strands of nucleic acids, which are typically less than 50 nucleotides, and provide a targeted approach to gene modification by binding to RNA and affecting splicing. Conventionally, the therapeutic development of oligonucleotides for many diseases has followed either classic antisense or siRNA approaches that rely on RNase H or RNA-induced silencing complex (RISC)-mediated pathways of transcript degradation (reviewed in [133]). These approaches have had some success in early-stage clinical trials of various disease areas, which has contributed to increased attention for antisense oligonucleotide therapy (reviewed in [133,134,135,136]). However, compared to classic antisense oligonucleotide-mediated mRNA transcript degradation, SSOs comprise a different type of antisense oligonucleotide therapy that alters normal splicing of the targeted transcript that may prove more versatile. Indeed, SSOs can be utilized to introduce a frameshift into the mature mRNA to introduce a premature termination codon that degrades transcripts through presumably nonsense-mediated mRNA decay to induce apoptosis of transformed cells and reduce tumor burden in an in vivo mouse model of mast cell neoplasia [137].

SSOs can promote the inclusion or removal of exons from mature mRNA. In the case of the latter, SSOs induce skipping of a specific exon in mature mRNA by binding to splicing sites in precursor-mRNA, resulting in a steric block of the spliceosome machinery proteins from binding to the site (Figure 2). Recent studies are also exploring the emerging phenomenon of cryptic splice site activation by antisense oligonucleotides, although this is, at present, a rare observation [138]. An advantage of SSOs is that chemical modifications to the backbones increase the stability of the antisense oligonucleotides and prevent degradation of pre-mRNA-SSO complexes by RNase H; if designed correctly, they will allow transcription of an altered mRNA to continue (reviewed in [139]). In some applications, exon exclusion or inclusion can correct aberrant splicing, or a frame-shift mutation to restore expression of a partially functional protein. For example, in Duchenne muscular dystrophy (DMD), exon skipping is utilized to reestablish the correct reading frame where a mutated exon that contains a frameshift is skipped to restore production of a partially functional dystrophin protein. This approach reduces the clinical severity of DMD (reviewed in [140]). A number of other genetic diseases may also benefit from SSO-based therapy, which are covered in other reviews [133,135,141,142].

**Figure 3:**
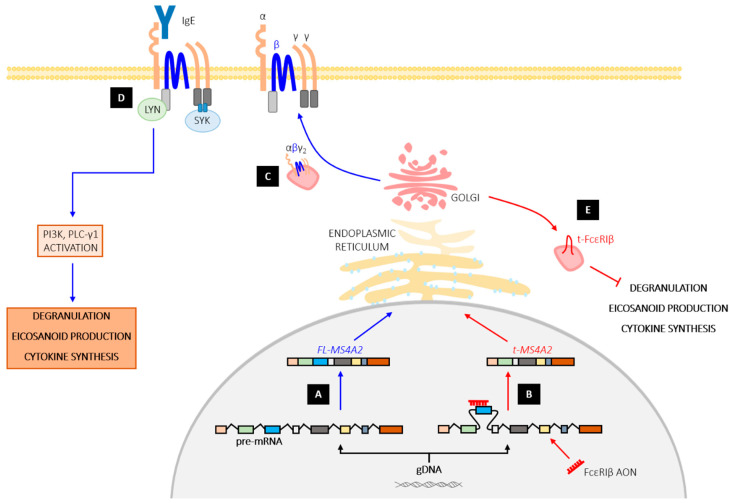
The functional effect of FcεRIβ antisense oligonucleotide (AON) treatment on mast cell activation. Blue arrows represent the path of full-length (FL) MS4A2 and FcεRIβ; red arrows represent the path of truncated (t)-MS4A2 and t-FcεRIβ, as a consequence of FcεRIβ exon skipping by FcεRIβ AONs. (**A**) MS4A2 pre-mRNA molecule undergoes normal splicing, resulting in transcription of FL-MS4A2 and translation of FcεRIβ. (**B**) In the presence of FcεRIβ AONs, exon 3 of MS4A2 pre-mRNA molecule is alternatively spliced, resulting in a truncated mature mRNA molecule, t-MS4A2. (**C**) FL-FcεRIβ forms complex with α and γ-subunits and traffics the receptor complex to the cell surface. (**D**) At the cell surface, FL-FcεRIβ stabilizes the receptor, enabling activation of FcεRI by antigen via crosslinking IgE antibodies, and subsequent proinflammatory cellular outcomes. (**E**) In contrast, t-MS4A2 is translated into t-FcεRIβ that lacks the first two transmembrane regions, rendering it incapable of trafficking FcεRIα to the plasma membrane.

To this end, FcεRIβ SSOs have been used to induce exon skipping and force the cell to preferentially produce the alternatively spliced t-FcεRIβ isoform [132]. Specifically, FcεRIβ SSOs have been used to target the splicing sites within exon 3. These SSOs yield a protein that resembles the alternative splice variant 3 of *MS4A2*, which prevents FcεRIβ from binding to and trafficking FcεRIα (Figure 3). In mouse bone marrow-derived mast cells (BMMCs) and the human mast cell line LAD2, FcεRIβ SSO treatment causes disproportionate expression of t-FcεRIβ and leads to a dose-dependent loss of surface expression of FcεRIα [132].

The loss of surface FcεRIα should impact IgE-mediated mast cell activation because, even in the presence of high levels of IgE, mast cells lacking surface FcεRIα will not have the capacity to bind IgE. Indeed, in BMMCs and, to a lesser extent, LAD2 cells, FcεRIβ SSO-induced loss of surface FcεRIα corresponds with inhibition of IgE-mediated degranulation [132] (Figure 3). Importantly, loss of surface FcεRIα is not directly proportional to the degree of inhibition of degranulation. This phenomenon is likely attributable to the small number of receptors required to trigger degranulation, since significant reductions in degranulation are only achieved with higher SSO concentrations where efficacy of exon skipping and loss of surface FcεRIα exceeds around 80% [132]. Thus, FcεRIβ SSOs may face similar challenges to therapies targeting serum IgE, such as omalizumab, as described above.

## 6. Conclusions

Targeting FcεRI and IgE-mediated mast cell and basophil activation has great therapeutic potential for allergic diseases. In addition to the established approaches that target IgE and binding of IgE to FcεRI, targeting FcεRI trafficking and signaling may also prove effective in IgE-mediated diseases. Identifying genes and proteins that play important roles in the FcεRI pathway is a critical step to finding novel targets for therapeutics. However, alternative splicing is often overlooked and splice variants can not only provide information for how pathways are regulated; once they are understood, the alternative splicing can also be utilized as a way to target those pathways. By altering the splicing of non-mutated but pathologically associated genes, the therapeutic potential of SSOs extends beyond diseases driven by genetic mutations. For allergic diseases and asthma, FcεRIβ is an ideal target for SSO therapy, since it has a characteristic role in IgE-mediated mast cell activation that is inhibited by SSO-mediated alternative splicing of the *MS4A2* gene.

## References

[1] Pawankar R. (2014). Allergic diseases and asthma: A global public health concern and a call to action. World Allergy Organ. J..

[2] Ribatti D. (2016). The development of human mast cells. An historical reappraisal. Exp. Cell Res..

[3] Reber L.L., Sibilano R., Mukai K., Galli S.J. (2015). Potential effector and immunoregulatory functions of mast cells in mucosal immunity. Mucosal Immunol..

[4] Galli S.J. (2016). The Mast Cell-IgE Paradox: From Homeostasis to Anaphylaxis. Am. J. Pathol..

[5] Sutton B.J., Davies A.M. (2015). Structure and dynamics of IgE-receptor interactions: FcεRI and CD23/FcεRII. Immunol. Rev..

[6] Burrows B., Martinez F.D., Halonen M., Barbee R.A., Cline M.G. (1989). Association of asthma with serum IgE levels and skin-test reactivity to allergens. N. Engl. J. Med..

[7] Gould H.J., Sutton B.J. (2008). IgE in allergy and asthma today. Nat. Rev. Immunol..

[8] Holgate S., Casale T., Wenzel S., Bousquet J., Deniz Y., Reisner C. (2005). The anti-inflammatory effects of omalizumab confirm the central role of IgE in allergic inflammation. J. Allergy Clin. Immunol..

[9] Lawrence M.G., Woodfolk J.A., Schuyler A.J., Stillman L.C., Chapman M.D., Platts-Mills T.A. (2017). Half-life of IgE in serum and skin: Consequences for anti-IgE therapy in patients with allergic disease. J. Allergy Clin. Immunol..

[10] MacGlashan D.W. (2007). Endocytosis, recycling, and degradation of unoccupied FcepsilonRI in human basophils. J. Leukoc. Biol..

[11] MacGlashan DJr Xia H.Z., Schwartz L.B., Gong J. (2001). IgE-regulated loss, not IgE-regulated synthesis, controls expression of FcepsilonRI in human basophils. J. Leukoc. Biol..

[12] Borkowski T.A., Jouvin M.H., Lin S.Y., Kinet J.P. (2001). Minimal requirements for IgE-mediated regulation of surface Fc epsilon RI. J. Immunol..

[13] Galli S.J., Tsai M., Piliponsky A.M. (2008). The development of allergic inflammation. Nature.

[14] Galli S.J., Tsai M. (2012). IgE and mast cells in allergic disease. Nat. Med..

[15] Kinet J.P. (1999). The high-affinity IgE receptor (Fc epsilon RI): From physiology to pathology. Annu. Rev. Immunol..

[16] Garman S.C., Kinet J.P., Jardetzky T.S. (1998). Crystal structure of the human high-affinity IgE receptor. Cell.

[17] Barata L.T., Ying S., Grant J.A., Humbert M., Barkans J., Meng Q., Durham S.R., Kay A.B. (1997). Allergen-induced recruitment of Fc epsilon RI+ eosinophils in human atopic skin. Eur. J. Immunol..

[18] Gounni A.S., Lamkhioued B., Ochiai K., Tanaka Y., Delaporte E., Capron A., Kinet J.P., Capron M. (1994). High-affinity IgE receptor on eosinophils is involved in defence against parasites. Nature.

[19] Gounni A.S., Lamkhioued B., Koussih L., Ra C., Renzi P.M., Hamid Q. (2001). Human neutrophils express the high-affinity receptor for immunoglobulin E (Fc epsilon RI): Role in asthma. FASEB J..

[20] Gounni A.S., Wellemans V., Yang J., Bellesort F., Kassiri K., Gangloff S., Guenounou M., Halayko A.J., Hamid Q., Lamkhioued B. (2005). Human airway smooth muscle cells express the high affinity receptor for IgE (Fc epsilon RI): A critical role of Fc epsilon RI in human airway smooth muscle cell function. J. Immunol..

[21] Hasegawa S., Pawankar R., Suzuki K., Nakahata T., Furukawa S., Okumura K., Ra C. (1999). Functional expression of the high affinity receptor for IgE (FcepsilonRI) in human platelets and its’ intracellular expression in human megakaryocytes. Blood.

[22] Maurer D., Fiebiger E., Reininger B., Wolff-Winiski B., Jouvin M.H., Kilgus O., Kinet J.P., Stingl G. (1994). Expression of functional high affinity immunoglobulin E receptors (Fc epsilon RI) on monocytes of atopic individuals. J. Exp. Med..

[23] Maurer D., Fiebiger S., Ebner C., Reininger B., Fischer G.F., Wichlas S., Jouvin M.H., Schmitt-Egenolf M., Kraft D., Kinet J.-P. (1996). Peripheral blood dendritic cells express Fc epsilon RI as a complex composed of Fc epsilon RI alpha- and Fc epsilon RI gamma-chains and can use this receptor for IgE-mediated allergen presentation. J. Immunol..

[24] Wang B., Rieger A., Kilgus O., Ochiai K., Maurer D., Födinger D., Kinet J.P., Stingl G. (1992). Epidermal Langerhans cells from normal human skin bind monomeric IgE via Fc epsilon RI. J. Exp. Med..

[25] Kraft S., Kinet J.-P. (2007). New developments in FcepsilonRI regulation, function and inhibition. Nat. Rev. Immunol..

[26] Garman S.C., Wurzburg B.A., Tarchevskaya S.S., Kinet J.P., Jardetzky T.S. (2000). Structure of the Fc fragment of human IgE bound to its high-affinity receptor Fc epsilonRI alpha. Nature.

[27] Jouvin M.H., Adamczewski M., Numerof R., Letourneur O., Vallé A., Kinet J.P. (1994). Differential control of the tyrosine kinases Lyn and Syk by the two signaling chains of the high affinity immunoglobulin E receptor. J. Biol. Chem..

[28] Kihara H., Siraganian R.P. (1994). Src homology 2 domains of Syk and Lyn bind to tyrosine-phosphorylated subunits of the high affinity IgE receptor. J. Biol. Chem..

[29] Gilfillan A.M., Tkaczyk C. (2006). Integrated signalling pathways for mast-cell activation. Nat. Rev. Immunol..

[30] Lin S., Cicala C., Scharenberg A.M., Kinet J.P. (1996). The Fc(epsilon)RIbeta subunit functions as an amplifier of Fc(epsilon)RIgamma-mediated cell activation signals. Cell.

[31] Rivera J., Gilfillan A.M. (2006). Molecular regulation of mast cell activation. J. Allergy Clin. Immunol..

[32] Hutchcroft J.E., Geahlen R.L., Deanin G.G., Oliver J.M. (1992). Fc epsilon RI-mediated tyrosine phosphorylation and activation of the 72-kDa protein-tyrosine kinase, PTK72, in RBL-2H3 rat tumor mast cells. Proc. Natl. Acad. Sci. USA.

[33] Shiue L., Zoller M.J., Brugge J.S. (1995). Syk is activated by phosphotyrosine-containing peptides representing the tyrosine-based activation motifs of the high affinity receptor for IgE. J. Biol. Chem..

[34] Schwartz S.L., Cleyrat C., Olah M.J., Relich P.K., Phillips G.K., Hlavacek W.S., Lidke K.A., Wilson B.S., Lidke D.S. (2017). Differential mast cell outcomes are sensitive to FcεRI-Syk binding kinetics. Mol. Biol. Cell..

[35] Travers T., Kanagy W.K., Mansbach R.A., Jhamba E., Cleyrat C., Goldstein B., Lidke D.S., Wilson B.S., Gnanakaran S. (2019). Combinatorial diversity of Syk recruitment driven by its multivalent engagement with FcεRIγ. MBoC.

[36] Mahajan A., Barua D., Cutler P., Lidke D.S., Espinoza F.A., Pehlke C., Grattan R., Kawakami Y., Tung C.S., Bradbury A.R. (2014). Optimal aggregation of FcεRI with a structurally defined trivalent ligand overrides negative regulation driven by phosphatases. ACS Chem. Biol..

[37] Wilson B.S., Oliver J.M., Lidke D.S. (2011). Spatio-temporal signaling in mast cells. Adv. Exp. Med Biol..

[38] Johnson S.A., Pleiman C.M., Pao L., Schneringer J., Hippen K., Cambier J.C. (1995). Phosphorylated immunoreceptor signaling motifs (ITAMs) exhibit unique abilities to bind and activate Lyn and Syk tyrosine kinases. J. Immunol..

[39] Sigalov A. (2005). Multi-chain immune recognition receptors: Spatial organization and signal transduction. Semin Immunol..

[40] Ashmole I., Duffy S.M., Leyland M.L., Morrison V.S., Begg M., Bradding P. (2012). CRACM/Orai ion channel expression and function in human lung mast cells. J. Allergy Clin. Immunol..

[41] Zhang S.L., Yu Y., Roos J., Kozak J.A., Deerinck T.J., Ellisman M.H., Stauderman K.A., Cahalan M.D. (2005). STIM1 is a Ca^2+^ sensor that activates CRAC channels and migrates from the Ca^2+^ store to the plasma membrane. Nature.

[42] Arthur G.K., Ehrhardt-Humbert L.C., Snider D.B., Jania C., Tilley S.L., Metcalfe D.D., Cruse G. (2020). The FcεRIβ homologue, MS4A4A, promotes FcεRI signal transduction and store-operated Ca^2+^ entry in human mast cells. Cell Signal..

[43] Lewis R.S. (2003). Calcium oscillations in T-cells: Mechanisms and consequences for gene expression. Biochem. Soc. Trans..

[44] Bradding P., Arthur G. (2016). Mast cells in asthma—State of the art. Clin. Exp. Allergy.

[45] Di Capite J., Parekh A.B. (2009). CRAC channels and Ca^2+^ signaling in mast cells. Immunol. Rev..

[46] Holowka D., Wilkes M., Stefan C., Baird B. (2016). Roles for Ca^2+^ mobilization and its regulation in mast cell functions: Recent progress. Biochem. Soc. Trans..

[47] Ma H.-T., Beaven M.A. (2009). Regulation of Ca^2+^ signaling with particular focus on mast cells. Crit. Rev. Immunol..

[48] Parekh A.B. (2009). Local Ca^2+^ influx through CRAC channels activates temporally and spatially distinct cellular responses. Acta Physiol..

[49] Kim M.-S., Rådinger M., Gilfillan A.M. (2008). The multiple roles of phosphoinositide 3-kinase in mast cell biology. Trends Immunol..

[50] Hartman M.-L., Lin S.-Y., Jouvin M.-H., Kinet J.-P. (2008). Role of the extracellular domain of Fc epsilon RI alpha in intracellular processing and surface expression of the high affinity receptor for IgE Fc epsilon RI. Mol. Immunol..

[51] Ra C., Jouvin M.H., Kinet J.P. (1989). Complete structure of the mouse mast cell receptor for IgE (Fc epsilon RI) and surface expression of chimeric receptors (rat-mouse-human) on transfected cells. J. Biol. Chem..

[52] Shin J.-S., Greer A.M. (2015). The role of FcεRI expressed in dendritic cells and monocytes. Cell Mol. Life Sci..

[53] Platzer B., Baker K., Vera M.P., Singer K., Panduro M., Lexmond W.S., Turner D., Vargas S.O., Kinet J.P., Maurer D. (2015). Dendritic cell-bound IgE functions to restrain allergic inflammation at mucosal sites. Mucosal Immunol..

[54] Greer A.M., Wu N., Putnam A.L., Woodruff P.G., Wolters P., Kinet J.-P., Shin J.S. (2014). Serum IgE clearance is facilitated by human FcεRI internalization. J. Clin. Invest..

[55] Ozpinar E.W., Frey A.L., Arthur G.K., Mora-Navarro C., Biehl A., Snider D.B., Cruse G., Freytes D.O. (2020). Dermal Extracellular Matrix-Derived Hydrogels as an In Vitro Substrate to Study Mast Cell Maturation. Tissue Eng. Part A.

[56] Kubo S., Nakayama T., Matsuoka K., Yonekawa H., Karasuyama H. (2003). Long term maintenance of IgE-mediated memory in mast cells in the absence of detectable serum IgE. J. Immunol..

[57] Beck L.A., Marcotte G.V., MacGlashan D., Togias A., Saini S. (2004). Omalizumab-induced reductions in mast cell Fce psilon RI expression and function. J. Allergy Clin. Immunol..

[58] Deza G., Bertolín-Colilla M., Sánchez S., Soto D., Pujol R.M., Gimeno R., Giménez-Arnau A.M. (2018). Basophil FcεRI expression is linked to time to omalizumab response in chronic spontaneous urticaria. J. Allergy Clin. Immunol..

[59] Dedaj R., Unsel L. (2019). Case study: A Combination of Mepolizumab and Omaluzimab injections for severe asthma. J. Asthma.

[60] Katsaounou P., Buhl R., Brusselle G., Pfister P., Martínez R., Wahn U., Bousquet J. (2019). Omalizumab as alternative to chronic use of oral corticosteroids in severe asthma. Respir. Med..

[61] Pelaia G., Gallelli L., Renda T., Romeo P., Busceti M.T., Grembiale R.D., Maselli R., Marsico S.A., Vatrella A. (2011). Update on optimal use of omalizumab in management of asthma. J. Asthma Allergy.

[62] Bousquet J., Humbert M., Gibson P.G., Kostikas K., Jaumont X., Pfister P., Nissen F. (2021). Real-world effectiveness of omalizumab in severe allergic asthma: A meta-analysis of observational studies. J. Allergy Clin. Immunol. Pract..

[63] Bachert C., Zhang L., Gevaert P. (2015). Current and future treatment options for adult chronic rhinosinusitis: Focus on nasal polyposis. J. Allergy Clin. Immunol..

[64] Nadeau K.C., Kohli A., Iyengar S., DeKruyff R.H., Umetsu D.T. (2012). Oral immunotherapy and anti-IgE antibody-adjunctive treatment for food allergy. Immunol. Allergy Clin. N. Am..

[65] Schneider L.C., Rachid R., LeBovidge J., Blood E., Mittal M., Umetsu D.T. (2013). A pilot study of omalizumab to facilitate rapid oral desensitization in high-risk peanut-allergic patients. J. Allergy Clin. Immunol..

[66] Iyengar S.R., Hoyte E.G., Loza A., Bonaccorso S., Chiang D., Umetsu D.T., Nadeau K.C. (2013). Immunologic effects of omalizumab in children with severe refractory atopic dermatitis: A randomized, placebo-controlled clinical trial. Int. Arch. Allergy Immunol..

[67] Kim D.H., Park K.Y., Kim B.J., Kim M.N., Mun S.K. (2013). Anti-immunoglobulin E in the treatment of refractory atopic dermatitis. Clin. Exp. Dermatol..

[68] Maurer M., Metz M., Brehler R., Hillen U., Jakob T., Mahler V., Pföhler C., Staubach P., Treudler R., Wedi B. (2018). Omalizumab treatment in patients with chronic inducible urticaria: A systematic review of published evidence. J. Allergy Clin. Immunol..

[69] Saini S.S., Bindslev-Jensen C., Maurer M., Grob J.-J., Bülbül Baskan E., Bradley M.S., Canvin J., Rahmaoui A., Georgiou P., Alpan O. (2015). Efficacy and safety of omalizumab in patients with chronic idiopathic/spontaneous urticaria who remain symptomatic on H1 antihistamines: A randomized, placebo-controlled study. J. Invest Dermatol..

[70] Yu M., Terhorst-Molawi D., Altrichter S., Hawro T., Chen Y.-D., Liu B., Song X.T., Zhao Z.T., Maurer M. (2021). Omalizumab in chronic inducible urticaria a real-life study of efficacy, safety, predictors of treatment outcome and time to response. Clin. Exp. Allergy.

[71] Just J., Thonnelier C., Bourgoin-Heck M., Mala L., Molimard M., Humbert M., STELLAIR Investigators (2021). Omalizumab Effectiveness in Severe Allergic Asthma with Multiple Allergic Comorbidities: A Post-Hoc Analysis of the STELLAIR Study. J. Asthma Allergy.

[72] Chan Y.-C., Ramadani F., Santos A.F., Pillai P., Ohm-Laursen L., Harper C.E., Fang C., Dodev T.S., Wu S.Y., Ying S. (2014). “Auto-anti-IgE”: Naturally occurring IgG anti-IgE antibodies may inhibit allergen-induced basophil activation. J. Allergy Clin. Immunol..

[73] MacGlashan D. (2009). Therapeutic efficacy of omalizumab. J. Allergy Clin. Immunol..

[74] Fahy J.V., Fleming H.E., Wong H.H., Liu J.T., Su J.Q., Reimann J., Fick R.B., Boushey H.A. (1997). The effect of an anti-IgE monoclonal antibody on the early- and late-phase responses to allergen inhalation in asthmatic subjects. Am. J. Respir. Crit. Care Med..

[75] Leung D.Y., Sampson H.A., Yunginger J.W., Burks A.W., Schneider L.C., Wortel C.H., Davis F.M., Hyun J.D., Shanahan W.R. (2003). Avon Longitudinal Study of Parents and Children Study Team. Effect of anti-IgE therapy in patients with peanut allergy. N. Engl. J. Med..

[76] Holgate S., Buhl R., Bousquet J., Smith N., Panahloo Z., Jimenez P. (2009). The use of omalizumab in the treatment of severe allergic asthma: A clinical experience update. Respir. Med..

[77] Gasser P., Tarchevskaya S.S., Guntern P., Brigger D., Ruppli R., Zbären N., Kleinboelting S., Heusser C., Jardetzky T.S., Eggel A. (2020). The mechanistic and functional profile of the therapeutic anti-IgE antibody ligelizumab differs from omalizumab. Nat. Commun..

[78] Maeyama K., Hohman R.J., Metzger H., Beaven M.A. (1986). Quantitative relationships between aggregation of IgE receptors, generation of intracellular signals, and histamine secretion in rat basophilic leukemia (2H3) cells. Enhanced responses with heavy water. J. Biol. Chem..

[79] Coleman J.W., Godfrey R.C. (1981). The number and affinity of IgE receptors on dispersed human lung mast cells. Immunology.

[80] Arm J.P., Bottoli I., Skerjanec A., Floch D., Groenewegen A., Maahs S., Owen C.E., Jones I., Lowe P.J. (2014). Pharmacokinetics, pharmacodynamics and safety of QGE031 (ligelizumab), a novel high-affinity anti-IgE antibody, in atopic subjects. Clin. Exp. Allergy.

[81] Gauvreau G.M., Arm J.P., Boulet L.-P., Leigh R., Cockcroft D.W., Davis B.E., Mayers I., FitzGerald J.M., Dahlen B., Killian K.J. (2016). Efficacy and safety of multiple doses of QGE031 (ligelizumab) versus omalizumab and placebo in inhibiting allergen-induced early asthmatic responses. J. Allergy Clin. Immunol..

[82] Maurer M., Giménez-Arnau A., Bernstein J.A., Chu C.Y., Danilycheva I., Hide M., Makris M., Metz M., Savic S., Sitz K. (2021). Sustained safety and efficacy of ligelizumab in patients with chronic spontaneous urticaria: A one-year extension study. Allergy.

[83] Khodoun M.V., Kucuk Z.Y., Strait R.T., Krishnamurthy D., Janek K., Lewkowich I., Morris S.C., Finkelman F.D. (2013). Rapid polyclonal desensitization with antibodies to IgE and FcεRIα. J. Allergy Clin. Immunol..

[84] Khodoun M.V., Morris S.C., Angerman E., Potter C., Schuman R., Wunderlich M., Maciag J.J., Sullivan Locker K.C., Mulloy J.C., Herr A.B. (2020). Rapid desensitization of humanized mice with anti-human FcεRIα monoclonal antibodies. J. Allergy Clin. Immunol..

[85] Saini S.S., MacGlashan D. (2002). How IgE upregulates the allergic response. Curr. Opin. Immunol..

[86] Molfetta R., Gasparrini F., Santoni A., Paolini R. (2010). Ubiquitination and endocytosis of the high affinity receptor for IgE. Mol. Immunol..

[87] Zhang K., Liu J., Truong T., Zukin E., Chen W., Saxon A. (2017). Blocking Allergic Reaction through Targeting Surface-Bound IgE with Low-Affinity Anti-IgE Antibodies. J. Immunol..

[88] Nakamura G.R., Starovasnik M.A., Reynolds M.E., Lowman H.B. (2001). A novel family of hairpin peptides that inhibit IgE activity by binding to the high-affinity IgE receptor. Biochemistry.

[89] Rossi M., Ruvo M., Marasco D., Colombo M., Cassani G., Verdoliva A. (2008). Anti-allergic properties of a new all-D synthetic immunoglobulin-binding peptide. Mol. Immunol..

[90] Zhou J.S., Sandomenico A., Severino V., Burton O.T., Darling A., Oettgen H.C., Ruvo M. (2013). An IgE receptor mimetic peptide (PepE) protects mice from IgE mediated anaphylaxis. Mol. Biosyst..

[91] Wiegand T.W., Williams P.B., Dreskin S.C., Jouvin M.H., Kinet J.P., Tasset D. (1996). High-affinity oligonucleotide ligands to human IgE inhibit binding to Fc epsilon receptor I. J. Immunol..

[92] Eggel A., Baumann M.J., Amstutz P., Stadler B.M., Vogel M. (2009). DARPins as bispecific receptor antagonists analyzed for immunoglobulin E receptor blockage. J. Mol. Biol..

[93] Eggel A., Baravalle G., Hobi G., Kim B., Buschor P., Forrer P., Shin J.S., Vogel M., Stadler B.M., Dahinden C.A. (2014). Accelerated dissociation of IgE-FcεRI complexes by disruptive inhibitors actively desensitizes allergic effector cells. J. Allergy Clin. Immunol..

[94] Delgado S.J., Dehmel S., Twisterling E., Wichmann J., Jonigk D., Warnecke G., Braubach P., Fieguth H.G., Wilkens L., Dahlmann F. (2020). Disruptive anti-IgE inhibitors prevent mast cell-dependent early airway response in viable atopic lung tissue. J. Allergy Clin. Immunol..

[95] Pennington L.F., Gasser P., Brigger D., Guntern P., Eggel A., Jardetzky T.S. (2021). Structure-guided design of ultrapotent disruptive IgE inhibitors to rapidly terminate acute allergic reactions. J. Allergy Clin. Immunol..

[96] Fong D.C., Malbec O., Arock M., Cambier J.C., Fridman W.H., Daëron M. (1996). Selective in vivo recruitment of the phosphatidylinositol phosphatase SHIP by phosphorylated Fc gammaRIIB during negative regulation of IgE-dependent mouse mast cell activation. Immunol. Lett..

[97] Ono M., Bolland S., Tempst P., Ravetch J.V. (1996). Role of the inositol phosphatase SHIP in negative regulation of the immune system by the receptor Fc(gamma)RIIB. Nature.

[98] Bolland S., Pearse R.N., Kurosaki T., Ravetch J.V. (1998). SHIP modulates immune receptor responses by regulating membrane association of Btk. Immunity.

[99] Malbec O., Attal J.-P., Fridman W.H., Daëron M. (2002). Negative regulation of mast cell proliferation by FcgammaRIIB. Mol. Immunol..

[100] Cemerski S., Chu S.Y., Moore G.L., Muchhal U.S., Desjarlais J.R., Szymkowski D.E. (2012). Suppression of mast cell degranulation through a dual-targeting tandem IgE-IgG Fc domain biologic engineered to bind with high affinity to FcγRIIb. Immunol. Lett..

[101] Ekoff M., Möller C., Xiang Z., Nilsson G. (2006). Coaggregation of FcepsilonRI with FcgammaRIIB Inhibits Degranulation but Not Induction of Bcl-2 Family Members A1 and Bim in Mast Cells. Allergy Asthma Clin. Immunol..

[102] Kepley C.L., Taghavi S., Mackay G., Zhu D., Morel P.A., Zhang K., Ryan J.J., Satin L.S., Zhang M., Pandolfi P.P. (2004). Co-aggregation of FcgammaRII with FcepsilonRI on human mast cells inhibits antigen-induced secretion and involves SHIP-Grb2-Dok complexes. J. Biol. Chem..

[103] Zellweger F., Gasser P., Brigger D., Buschor P., Vogel M., Eggel A. (2017). A novel bispecific DARPin targeting FcγRIIB and FcεRI-bound IgE inhibits allergic responses. Allergy.

[104] Barker M.D., Liddle J., Atkinson F.L., Wilson D.M., Dickson M.C., Ramirez-Molina C., Lewis H., Davis R.P., Somers D.O., Neu M. (2018). Discovery of potent and selective Spleen Tyrosine Kinase inhibitors for the topical treatment of inflammatory skin disease. Bioorg. Med. Chem. Lett..

[105] Lin W., Su F., Gautam R., Wang N., Zhang Y., Wang X. (2018). Raf kinase inhibitor protein negatively regulates FcεRI-mediated mast cell activation and allergic response. Proc. Natl. Acad. Sci. USA.

[106] Ramirez Molina C., Falkencrone S., Skov P.S., Hooper-Greenhill E., Barker M., Dickson M.C. (2019). GSK2646264, a spleen tyrosine kinase inhibitor, attenuates the release of histamine in ex vivo human skin. Br. J. Pharmacol..

[107] Evans E.K., Gardino A.K., Kim J.L., Hodous B.L., Shutes A., Davis A., Zhu X.J., Schmidt-Kittler O., Wilson D., Wilson K. (2017). A precision therapy against cancers driven by KIT/PDGFRA mutations. Sci. Transl. Med..

[108] Koziol-White C.J., Jia Y., Baltus G.A., Cooper P.R., Zaller D.M., Crackower M.A., Sirkowski E.E., Smock S., Northrup A.B., Himes B.E. (2016). Inhibition of spleen tyrosine kinase attenuates IgE-mediated airway contraction and mediator release in human precision cut lung slices. Br. J. Pharmacol..

[109] Matsubara S., Li G., Takeda K., Loader J.E., Pine P., Masuda E.S., Miyahara N., Miyahara S., Lucas J.J., Dakhama A. (2006). Inhibition of spleen tyrosine kinase prevents mast cell activation and airway hyperresponsiveness. Am. J. Respir. Crit. Care Med..

[110] Penton P.C., Wang X., Amatullah H., Cooper J., Godri K., North M.L., Khanna N., Scott J.A., Chow C.W. (2013). Spleen tyrosine kinase inhibition attenuates airway hyperresponsiveness and pollution-induced enhanced airway response in a chronic mouse model of asthma. J. Allergy Clin. Immunol..

[111] Adra C.N., Mao X.Q., Kawada H., Gao P.S., Korzycka B., Donate J.L., Shaldon S.R., Coull P., Dubowitz M., Enomoto T. (1999). Chromosome 11q13 and atopic asthma. Clin. Genet..

[112] Hill M.R., Cookson W.O. (1996). A new variant of the beta subunit of the high-affinity receptor for immunoglobulin E (Fc epsilon RI-beta E237G): Associations with measures of atopy and bronchial hyper-responsiveness. Hum. Mol. Genet..

[113] Kim Y.-K., Park H.-W., Yang J.-S., Oh S.-Y., Chang Y.-S., Shin E.-S., Lee J.E., Kim S., Gho Y.S., Cho S.H. (2007). Association and functional relevance of E237G, a polymorphism of the high-affinity immunoglobulin E-receptor beta chain gene, to airway hyper-responsiveness. Clin. Exp. Allergy..

[114] Laprise C., Boulet L.P., Morissette J., Winstall E., Raymond V. (2000). Evidence for association and linkage between atopy, airway hyper-responsiveness, and the beta subunit Glu237Gly variant of the high-affinity receptor for immunoglobulin E in the French-Canadian population. Immunogenetics.

[115] Zhang X., Zhang W., Qiu D., Sandford A., Tan W.C. (2004). The E237G polymorphism of the high-affinity IgE receptor beta chain and asthma. Ann. Allergy Asthma Immunol..

[116] Ishizawa M., Shibasaki M., Yokouchi Y., Noguchi E., Arinami T., Yamakawa-Kobayashi K., Matsui A., Hamaguchi H. (1999). No association between atopic asthma and a coding variant of Fc epsilon R1 beta in a Japanese population. J. Hum. Genet..

[117] Simon Thomas N., Wilkinson J., Lonjou C., Morton N.E., Holgate S.T. (2000). Linkage analysis of markers on chromosome 11q13 with asthma and atopy in a United Kingdom population. Am. J. Respir. Crit. Care Med..

[118] Zhu S., Chan-Yeung M., Becker A.B., Dimich-Ward H., Ferguson A.C., Manfreda J., Watson W.T., Paré P.D., Sandford A.J. (2000). Polymorphisms of the IL-4, TNF-alpha, and Fcepsilon RIbeta genes and the risk of allergic disorders in at-risk infants. Am. J. Respir. Crit. Care Med..

[119] Donnadieu E., Jouvin M.H., Kinet J.P. (2000). A second amplifier function for the allergy-associated Fc(epsilon)RI-beta subunit. Immunity.

[120] Furumoto Y., Hiraoka S., Kawamoto K., Masaki S., Kitamura T., Okumura K., Ra C. (2000). Polymorphisms in FcepsilonRI beta chain do not affect IgE-mediated mast cell activation. Biochem. Biophys. Res. Commun..

[121] Kim S.-H., Bae J.-S., Holloway J.W., Lee J.-T., Suh C.-H., Nahm D.-H., Park H.S. (2006). A polymorphism of MS4A2 (- 109T > C) encoding the beta-chain of the high-affinity immunoglobulin E receptor (FcepsilonR1beta) is associated with a susceptibility to aspirin-intolerant asthma. Clin. Exp. Allergy.

[122] Nishiyama C., Akizawa Y., Nishiyama M., Tokura T., Kawada H., Mitsuishi K., Hasegawa M., Ito T., Nakano N., Okamoto A. (2004). Polymorphisms in the Fc epsilon RI beta promoter region affecting transcription activity: A possible promoter-dependent mechanism for association between Fc epsilon RI beta and atopy. J. Immunol..

[123] Yang H.-J., Zheng L., Zhang X.-F., Yang M., Huang X. (2014). Association of the MS4A2 gene promoter C-109T or the 7th exon E237G polymorphisms with asthma risk: A meta-analysis. Clin. Biochem..

[124] Baralle F.E., Giudice J. (2017). Alternative splicing as a regulator of development and tissue identity. Nat. Rev. Mol. Cell Biol..

[125] Will C.L., Lührmann R. (2011). Spliceosome structure and function. Cold Spring Harb. Perspect. Biol..

[126] Kraft S., Rana S., Jouvin M.-H., Kinet J.-P. (2004). The role of the FcepsilonRI beta-chain in allergic diseases. Int. Arch. Allergy Immunol..

[127] Singleton T.E., Platzer B., Dehlink E., Fiebiger E. (2009). The first transmembrane region of the beta-chain stabilizes the tetrameric Fc epsilon RI complex. Mol. Immunol..

[128] Donnadieu E., Jouvin M.-H., Rana S., Moffatt M.F., Mockford E.H., Cookson W.O., Kinet J.P. (2003). Competing functions encoded in the allergy-associated F(c)epsilonRIbeta gene. Immunity.

[129] Cruse G., Kaur D., Leyland M., Bradding P. (2010). A novel FcεRIβ-chain truncation regulates human mast cell proliferation and survival. FASEB J..

[130] Cruse G., Beaven M.A., Ashmole I., Bradding P., Gilfillan A.M., Metcalfe D.D. (2013). A truncated splice-variant of the FcεRIβ receptor subunit is critical for microtubule formation and degranulation in mast cells. Immunity.

[131] Nishida K., Yamasaki S., Ito Y., Kabu K., Hattori K., Tezuka T., Nishizumi H., Kitamura D., Goitsuka R., Geha R.S. (2005). Fc{epsilon}RI-mediated mast cell degranulation requires calcium-independent microtubule-dependent translocation of granules to the plasma membrane. J. Cell Biol..

[132] Cruse G., Yin Y., Fukuyama T., Desai A., Arthur G.K., Bäumer W., Beaven M.A., Metcalfe D.D. (2016). Exon skipping of FcεRIβ eliminates expression of the high-affinity IgE receptor in mast cells with therapeutic potential for allergy. Proc. Natl. Acad. Sci. USA.

[133] Lundin K.E., Gissberg O., Smith C.I.E. (2015). Oligonucleotide Therapies: The Past and the Present. Hum. Gene Ther..

[134] Keinath M.C., Prior D.E., Prior T.W. (2021). Spinal Muscular Atrophy: Mutations, Testing, and Clinical Relevance. Appl. Clin. Genet..

[135] Potaczek D.P., Garn H., Unger S.D., Renz H. (2016). Antisense molecules: A new class of drugs. J. Allergy Clin. Immunol..

[136] Schneider A.-F.E., Aartsma-Rus A. (2020). Developments in reading frame restoring therapy approaches for Duchenne muscular dystrophy. Expert Opin. Biol. Ther..

[137] Snider D.B., Arthur G.K., Falduto G.H., Olivera A., Ehrhardt-Humbert L.C., Smith E., Smith C., Metcalfe D.D., Cruse G. (2021). Targeting KIT by frameshifting mRNA transcripts as a therapeutic strategy for aggressive mast cell neoplasms. Mol. Ther..

[138] Ham K.A., Keegan N.P., McIntosh C.S., Aung-Htut M.T., Zaw K., Greer K., Fletcher S., Wilton S.D. (2021). Induction of cryptic pre-mRNA splice-switching by antisense oligonucleotides. Sci. Rep..

[139] Havens M.A., Hastings M.L. (2016). Splice-switching antisense oligonucleotides as therapeutic drugs. Nucleic Acids Res..

[140] Nakamura A. (2017). Moving towards successful exon-skipping therapy for Duchenne muscular dystrophy. J. Hum. Genet..

[141] Aslesh T., Yokota T. (2020). Development of Antisense Oligonucleotide Gapmers for the Treatment of Dyslipidemia and Lipodystrophy. Methods Mol. Biol..

[142] Aoki Y., Wood M.J.A. (2021). Emerging Oligonucleotide Therapeutics for Rare Neuromuscular Diseases. J. Neuromuscul. Dis..

